# Two new species of *Olecryptotendipes* Zorina, 2007 from China (Diptera, Chironomidae)

**DOI:** 10.3897/zookeys.208.3299

**Published:** 2012-07-17

**Authors:** Chun-Cai Yan, Xin-Hua Wang, Wen-Jun Bu

**Affiliations:** 1 Tianjin Key Laboratory of Cyto-Genetical and Molecular Regulation, College of Life Sciences, Tianjin Normal University, Tianjin 300387, China;; 2 College of Life Sciences, Nankai University, Tianjin 300071, China

**Keywords:** Chironomidae, *Olecryptotendipes*, new species, key, China

## Abstract

Two new species, *Olecryptotendipes exilis* **sp. n.** and *Olecryptotendipes melasmus* **sp. n.** are described and illustrated as males and Chinese males of *Olecryptotendipes lenzi* are re-examined. A key to all known males of *Olecryptotendipes* is provided.

## Introduction

Based on the morphology of the males in the Russian Far East, *Cryptotendipes lenzi* Zorina, 2001 and *Cryptotendipes secundus* Zorina, 2003 were described by Zorina (2001, 2003). Zorina (2007) collected the larvae and pupae of *Cryptotendipes lenzi*, and showed the two species should be placed in a new genus, named *Olecryptotendipes* Zorina, 2007. The type species is *Cryptotendipes lenzi* Zorina, 2001. To date, the genus only contains the two aforementioned species. Yanet al. (2005) recorded *Cryptotendipes lenzi* from Xinjiang Autonomous Region in China.

The males of *Olecryptotendipes* are characterized by Y-shaped anal tergite bands; posterior part of tergite IX elongated with setae; superior volsella with sclerotized part and membranous ridge, with dorsal and ventral setae, microtrichia absent or present ventrally and weak inferior volsella (Zorina 2007). For the diagnosis of pupa and larva, refer to Zorina (2007).

In the present paper, two new species are recorded. Prof. Ole Sæther and Dr. M. Spies have checked the specimens. The two new species don’t belong to the genera *Cryptotendipes* Lenz, 1941 and *Chernovskiia* Sæther, 1977 because of the presence of sclerotized superior volsella and lobate inferior volsella. In addition, the species of the genus *Cryptotendipes* lack an inferior volsella and the margin of the gonostylus is usually with an expansion. Species of the genus *Chernovskiia* are also without lobate inferior volsella, but with foot-shaped superior volsella, which also present in species of genera *Paracladopelma* Harnisch, 1923 and *Beckidia* Kieffer, 1913. The Y-shaped anal tergite bands (Figs 2, 8), the shoulder-like margin of tergite IX (Figs 2, 8), the sclerotized superior volsella (Figs 4, 10) and lobate inferior volsella (Figs 3, 9) show them to belong to the genus *Olecryptotendipes* (O. Sæther and M. Spies, pers. comm.). However, we have no specimens of larvae and pupae which are important to place the species properly.

**Map 1.**
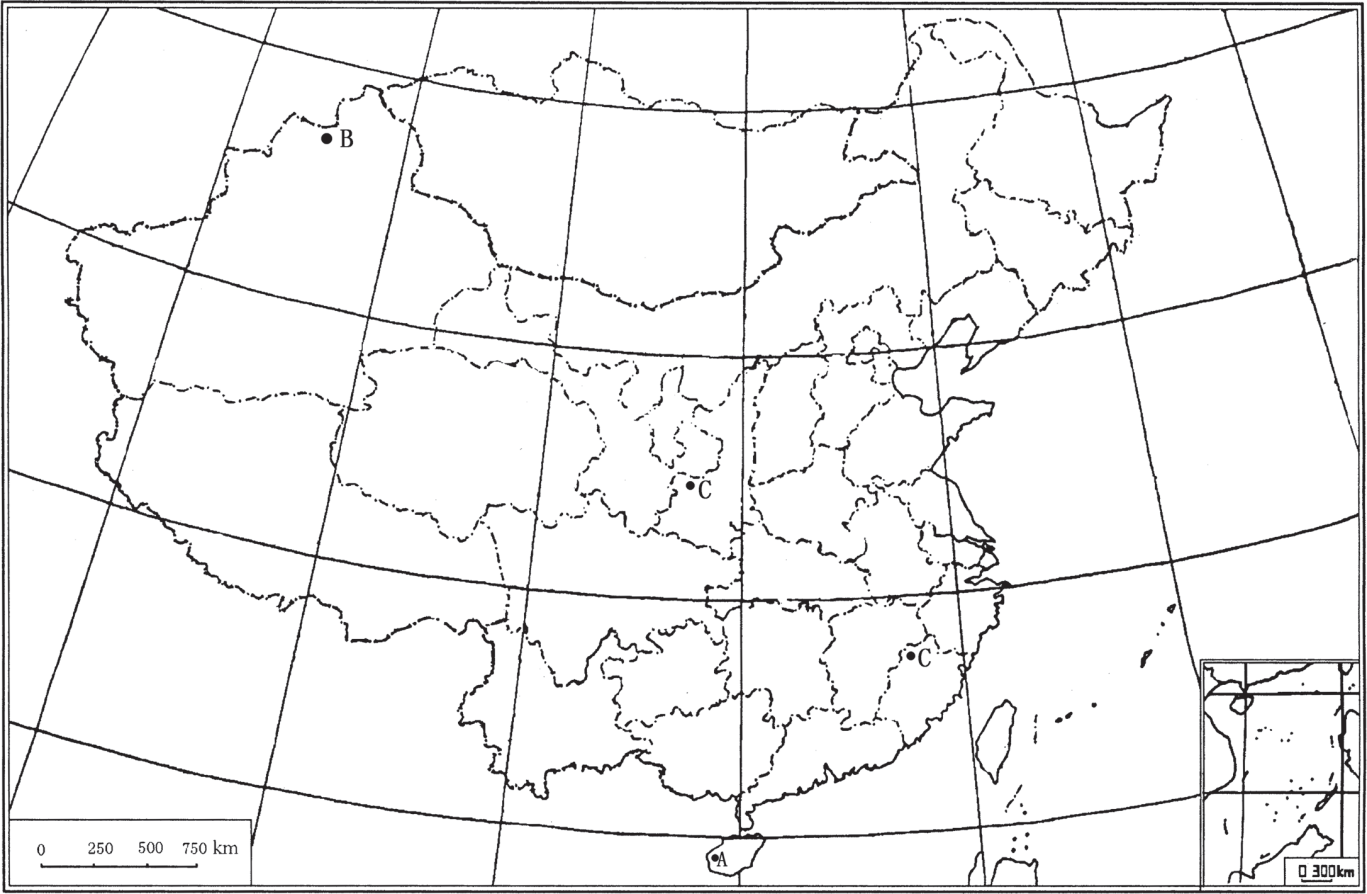
Distribution in China for the genus *Olecryptotendipes*. **A** *Olecryptotendipes exilis* sp. n. **B** *Olecryptotendipes lenzi* Zorina **C** *Olecryptotendipes melasmus* sp. n.

The larvae of *Olecryptotendipes* inhabit sandy substrate in rivers (Zorina 2007). The Chinese specimens were collected from temperate zones and subtropical mountain areas in Palaearctic and Oriental China (Map 1).

Two new species, *Olecryptotendipes exilis* sp. n. and *Olecryptotendipes melasmus* sp. n., are described and illustrated based on material from China and a key to the males of *Olecryptotendipes* is provided.

## Material and methods

The terminology follows Sæther (1980) with the additions and corrections given by Sæther (1990). The material examined was mounted on slides in Canada balsam following the procedure outlined by Sæther (1969).

The type material and other material studied are housed in the College of Life Science, Department of Biology, Nankai University, Tianjin, China (BDN).

## Taxonomy

### Amended generic diagnosis

Based on Zorina (2007) and the Chinese specimens, we amend the generic diagnosis. The following combination of characters will separate the genus *Olecryptotendipes* from other members of the *Harnischia* complex: Total length 2.7–3.6 mm; AR 1.83-2.24; frontal tubercles absent; anal tergite bands of Y-shaped; posterior margin of tergite IX with caudolateral shoulders; anal point parallel-sided or widest at about apical 1/3; superior volsella consisting of sclerotized part, dorsal and ventral setae present; inferior volsella with a weak blunt caudal projection, covered with microtrichia; gonostylus parallel-sided or slender to apex, inner margin without expansion.

#### Olecryptotendipes exilis

sp. n.

urn:lsid:zoobank.org:act:843E24C2-A4C2-4BDF-B681-582F6CBF1692

http://species-id.net/wiki/Olecryptotendipes_exilis

Figs 1–4

##### Diagnostic characters.

The species is separated by the slender posterolateral projection of the superior volsella and the lobate inferior volsella, the posterolateral weak lobes of the anal tergite, and the parallel-sided anal point.

##### Description.

Male imago (n=2). Total length 2.70−2.94 mm. Wing length 1.38−1.50 mm. Total length / wing length 1.96. Wing length / length of profemur 1.92−2.08.

Coloration. Thorax and legs dark brown. Abdomen with tergite I–V yellowish brown and tergite VI–VIII and hypopygium dark brown.

Head. AR 1.97−2.02. Ultimate flagellomere 590−600 mm long. Frontal tubercles absent. Temporal 13−15 setae, including 4 inner verticals, 5−6 outer verticals and 4−5 postorbitals. Clypeus with 13 setae. Tentorium 103−110 mm long, 28−30 mm wide. Palpomere lengths (in mm): 36−38, 38−40, 118−120, 140−145, 203−210. Palp segment 5^th^ / 3^rd^ 1.72−1.75.

Thorax. Antepronotals with 6−7 setae, dorsocentrals 8−10, acrostichals 8, prealars 3. Scutellum with 12−13 setae.

**Figures 1–4.**
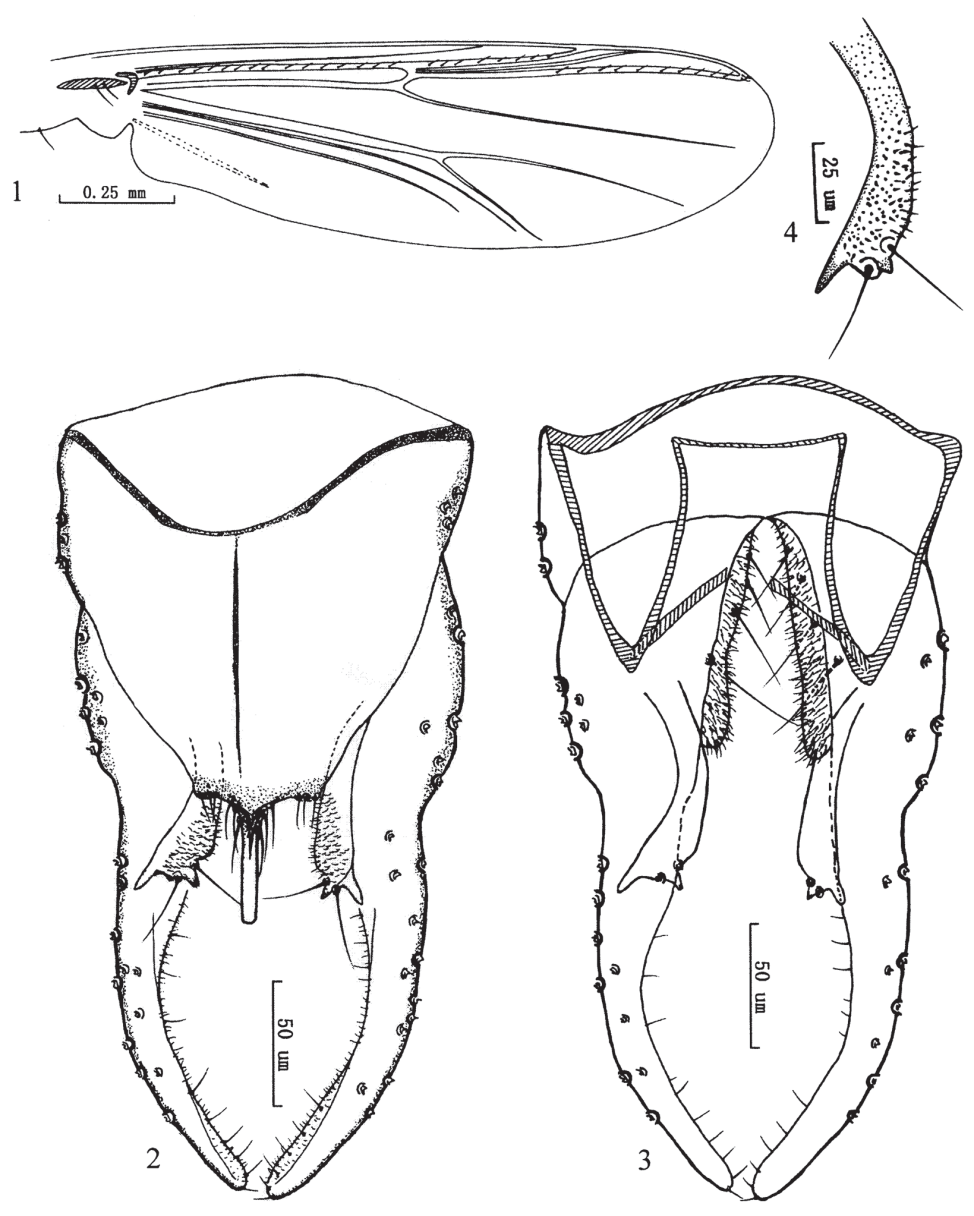
*Olecryptotendipes exilis* sp. n., male. **1** wing. **2** hypopygium (dorsal view ) **3** hypopygium (ventral view) **4** superior volsella.

Wing (Fig. 1). VR 1.19−120, R with 16−18 setae, R_1_ with 8−10 setae, R_4+5_ with 10−11 setae. Brachiolum 2 setae. Squama with 2−3 setae.

Legs. Front tibia with 3 subapical setae, 113−120, 138−144 and 141−150 µm long, spurs of mid tibia 30−35 and 37−46 µm long excluding comb, comb with 32−36 teeth, 10 µm long; spurs of hind tibia 28−30 and 38−47 µm long excluding comb, comb with 42−48 teeth, 10−12 µm long. Ta_1_ of mid legs with only 1 sensilla chaetica, sensilla chaetica absent in hind legs. Lengths (in µm) and proportions of legs as in Table 1.

**Table 1.** Lengths (µm) and proportion of legs of *Olecryptotendipes exilis* sp. n., male (n=2).

	**Fe**	**ti**	**ta_1_**	**ta_2_**	**ta_3_**	**ta_4_**	**ta_5_**	**LR**
p_1_	680−720	500−520	920−1080	490−515	380−410	310−320	150−160	1.84−1.08
p_2_	630−650	530−550	340−365	190−220	120−135	70−80	60−70	0.64−0.66
p_3_	700−720	710−725	480−495	250−260	220−230	110−115	90−95	0.68

Hypopygium (Figs 2−4). Tergite IX with weak lobes bearing 3−4 setae at each side of base of anal point. Laterosternite IX with 3 setae. Anal point 45−50 mm long, 5−6 mm wide, originating from caudal margin of anal tergite, completely parallel-side. Anal tergite bands Y-shaped. Phallapodeme 75−82 mm long. Transverse sternapodeme 56−60 mm long. Superior volsella (Fig. 4) slightly curved, with apical, partially sclerotized beak-like protrusion and slender spur-like posterolateral projection, bearing two long setae beside the beak-like protrusion, and covered with microtrichia in inner parts. Inferior volsella with a moderately blunt caudal projection, covered with microtrichia, and not reaching beyond margin of anal tergite. Gonocoxite 98−104 mm long, with 4 strong inner marginal setae. Gonostylus 168−175 mm long, slightly swollen at base, curved medially, moderately slender to apex, bearing 17−20 setae along inner margin. HR 0.58−0.59; HV 1.61−1.68.

##### Type material.

Holotype ♂ (BDN No. 1291). CHINA: Hainan Province, Ledong Li Nationality Autonomous County, Jianfengling Nature Conservation area, 18°14'45.96"N, 109°30'42.69"E, 21.iv.1985, X. Wang. Paratype 1♂ (BDN No. 03578), same data as holotype.

##### Etymology. 

From Latin *exilis*, slender, in reference to the slender posterolateral projections of superior volsella.

##### Distribution.

The species was collected in a subtropical mountain area in Hainan province in Oriental China.

#### Olecryptotendipes lenzi

(Zorina)

http://species-id.net/wiki/Olecryptotendipes_lenzi

Cryptotendipes lenzi Zorina 2001: 31; Zorina 2007: 350; Yan et al. 2005: 4

##### Specimens examined.

China, Xinjiang Autonomous Region: 13♂♂, Kaba Altay, Baihualin Nature Conservation area, 48°03'39.05"N, 86°25'7.04"E, 15−16.vii.2002, Tang HQ, sweep net.

##### Diagnostic characters.

Based on Zorina (2001) and Yan et al. (2005), the species is easily distinguished from the other species of the genus by having shoulder-like posterior margin of tergite IX and digitiform superior volsella with a longitudinal keel.

##### Distribution.

This species is distributed in the Russian Far East and northwestern China.

#### Olecryptotendipes melasmus sp. n.

urn:lsid:zoobank.org:act:A177BD2D-75F5-43F5-A269-E0FA3D9B22DC

http://species-id.net/wiki/Olecryptotendipes_melasmus

Figs 5–10

##### Diagnosis.

The species can be separated by the blackish brown spots on thorax and legs, the distally swollen anal point and the gonostylus with basal weak expansion.

##### Description.

Male imago (n=2). Total length 3.13–3.60 mm. Wing length 1.50–1.88 mm. Total length / wing length 1.91–2.09. Wing length / length of profemur 2.05–2.14.

**Figures 5–10.**
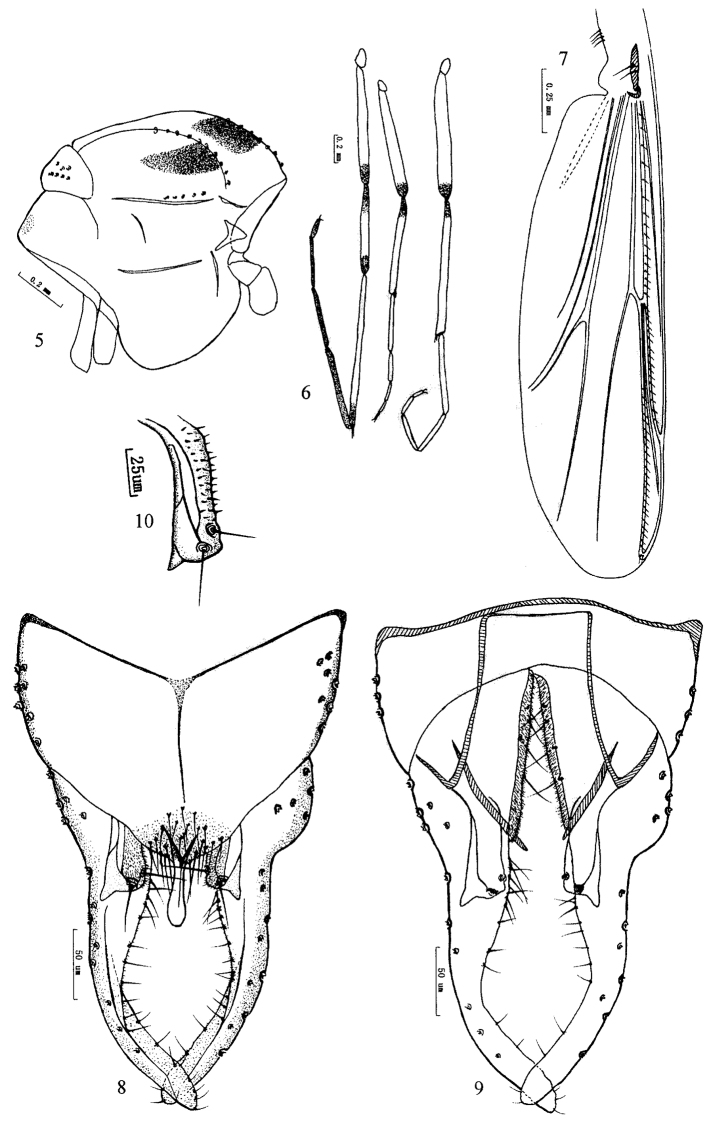
*Olecryptotendipes melasmus* sp. n., male. **5** thorax **6** leg **7** wing. **8** hypopygium (dorsal view) **9** hypopygium (ventral view) **10** superior volsella.

Coloration. Thorax (Fig. 5) yellowish brown, with median black brown vittae. Femur of front leg yellowish green with distal parts dark brown, tibia dark brown except for median parts yellowish green, tarsi dark brown with basal 3/4 of ta_1_ yellowish green; femora and tibia of mid and hind legs yellowish green with distal parts of femora and basal parts of tibia dark brown, ta_1_ to ta_5_ lightly brown (Fig. 6). Abdomen yellowish green to brown, with tergite I–V yellowish green, tergite VI–VIII and hypopygium yellowish brown.

Head. AR 1.94–2.24. Ultimate flagellomere 620–760 mm long. Frontal tubercles absent. Temporal 13–15 setae, including 4–5 inner verticals, 4–6 outer verticals and 4–5 postorbitals. Clypeus with 13–21 setae. Tentorium 113–120 mm long, 27–37 mm wide. Palpomere lengths (in mm): 37–40, 39–45, 133–172, 133–158, 193–223. Palp segment 5^th^ / 3^rd^ 1.30–1.45.

Thorax. Antepronotum with 2 setae, dorsocentrals 8–10, acrostichals 4–7, prealars 4–5. Scutellum with 12–15 setae.

Wing (Fig. 7). VR 1.15–1.20, R with 12–16 setae, R_1_ with 11–16 setae, R_4+5_ with 18–25 setae. Brachiolum 2 setae. Squama with 3–4 setae.

Legs. Front tibia with 3 subapical setae, 113–135, 133–150 and 145–170 µm long, spurs of middle tibia 28–30 and 35–37 µm long excluding comb, comb with 22–34 teeth, 10–11 µm long; spurs of hind tibia 30–32 and 42–43 µm long excluding comb, comb with 34–45 teeth, 10–12 µm long. Sensilla chaetica of mid and hind legs absent. Lengths (in µm) and proportions of legs as in Table 2.

**Table 2.** Lengths (µm) and proportion of legs of *Olecryptotendipes melasmus* sp. n., male (n=2).

	**Fe**	**Ti**	**ta_1_**	**ta_2_**	**ta_3_**	**ta_4_**	**Ta_5_**	**LR**
P_1_	730–880	500–650	1100 (1)	580 (1)	430 (1)	350 (1)	150 (1)	1.69 (1)
P_2_	640–800	530–700	330–410	180–220	120–150	70–85	60	0.59–0.62
P_3_	760–910	720–940	470–580	280–340	220–270	120–140	70–80	0.62–0.65

*Hypopygium* (Figs 8–10). Tergite IX with weak shoulder-like corners, bearing 13–20 setae at base of anal point. Laterosternite IX with 4–7 setae. Anal point originating from anterior of caudal margin of anal tergite in dorsal view, constricted basally, swollen apically, 43–50 mm long, 8–10 mm wide at base, 9–12 mm wide at apex. Anal tergite bands Y-shaped. Phallapodeme 68–80 mm long. Transverse sternapodeme 44–70 mm long. Superior volsella (Fig. 10) curved basally, straight distally, with large posterolateral projection, which is constricted medially forming a sharp angle, longitudinal membranous ridge present, bearing two long setae in distinct pits; covered with microtrichia in inner parts of superior volsella. Inferior volsella with reduced lobate caudal projection. Gonocoxite 85–100 mm long, with 4 strong inner marginal setae. Gonostylus 150–190 mm long, slightly swollen at base, concave medially, with rounded apex, bearing 8–20 setae along basal inner margin, and 10–14 setae along distal inner margin. HR 0.53–0.57; HV 1.89–2.09.

##### Type material.

Holotype ♂ (BDN No. 04250). CHINA: Shaanxi Province, Baoji City, Feng County, Qinling, Dongyu, 33°54'42.03"N, 106°31'21.10"E, 30. vii. 1994, sweep net, W. Bu. Paratype (BDN No. 20598). CHINA: 1♂, Fujian Province, Jianning County, 26°49'51.25"N, 116°50'45.90"E, 25. ix. 2002, light trap, Z. Liu.

##### Etymology.

From Greek, *melasma*, spot, in reference to the dark brown spots on the thorax and legs.

##### Distribution.

The species is known from Palaearctic and Oriental China (Shaanxi Province; Fujian Province).

##### Systematic remarks.

Male diagnosis: Based on the variation in Chinese material (*Olecryptotendipes exilis* sp. n., *Olecryptotendipes melasmus* sp. n.), the generic description given by Zorina (2007) should be emended as follows: “Antennal ratio 1.83–2.06” should be changed to “Antennal ratio 1.83–2.24”. “Total length 3.0–3.5 mm” should be emended to “Total length 2.7–3.6 mm.”

Based on the description and figures of Zorina (2003), posterior margin of tergite IX of *Olecryptotendipes secundus* not elongate but with caudolateral shoulders bearing 4 setae, which seem to *Olecryptotendipes exilis* sp. n. The gonostylus of *Olecryptotendipes lenzi* and *Olecryptotendipes secundus* nearly parallel-sided, but which is slender to apex of the two new species. The species *Olecryptotendipes secundus* only with sclerotized superior volsella and without membranous ridges, it seems to *Olecryptotendipes exilis* sp. n. with sclerotized beak-like protrusion. From the above, the generic characters of *Olecryptotendipes* should be emended as follows: “Posterior margin of tergite IX with caudolateral shoulders; gonostylus parallel-sided or slender to apex; superior volsella consisting of sclerotized part, dorsal and ventral setae present.”

### Key to males of the genus *Olecryptotendipes* in the world

**Table:** 

1	Acrostichals absent; R and R_1_ without setae; superior volsella lacking microtrichia ventrally	*Olecryptotendipes secundus* (Zorina)
–	Acrostichals present; R and R_1_ with setae; superior volsella with microtrichia ventrally	2
2	Anal point swollen distally; thorax with dark brown spots	*Olecryptotendipes melasmus* sp. n.
–	Anal point parallel-sided, thorax without dark brown spots	3
3	Inferior volsella absent, gonostylus parallel-sided	*Olecryptotendipes lenzi* (Zorina)
–	Inferior volsella lobe-like, gonostylus swollen at base, moderately slender to apex	*Olecryptotendipes exilis* sp. n.

## Supplementary Material

XML Treatment for Olecryptotendipes exilis

XML Treatment for Olecryptotendipes lenzi

XML Treatment for Olecryptotendipes melasmus
